# *Millettia speciosa* and By-Products: A Comprehensive Review of Chemical Composition, Bioactivities, Safety, and Industrial Applications

**DOI:** 10.3390/foods14122035

**Published:** 2025-06-09

**Authors:** Juntai Chen, Wenyong Lou

**Affiliations:** Lab of Applied Biocatalysis, School of Food Science and Engineering, South China University of Technology, No. 381 Wushan Road, Guangzhou 510640, China; 15302223985@163.com

**Keywords:** *Millettia speciosa*, phytochemical analysis, by-products, functional components, positive health outcomes, health risk assessment, industrial applications

## Abstract

*Millettia speciosa* (*MS*) is a traditional medicinal and edible plant with notable nutritional value and a wide range of biological activities. Recent studies have explored its chemical composition and pharmacological effects, particularly focusing on the potential utilization of its by-products. However, a lack of integrated analysis has limited a comprehensive understanding of its functional value and industrial relevance. This review provides a critical overview of the major bioactive components of MS, such as flavonoids, phenolic acids, alkaloids, saponins, and polysaccharides, and highlights their antioxidant, lipid-lowering, antimicrobial, antidepressant, and immune-regulatory properties. In addition, the nutritional aspects of MS, including its protein, amino acid, and mineral contents, are discussed in detail. Safety evaluations based on existing toxicological and pharmacokinetic studies are also summarized. Furthermore, this work outlines the current and emerging applications of MS and its by-products in key industries: in the food sector as functional ingredients and nutritional supplements, in agriculture as biofertilizers and animal feed additives, and in cosmetics for their antioxidative and skin-protective effects. Finally, the review identifies current challenges and prospects for the industrial development of MS-based products across multiple domains.

## 1. Introduction

*Millettia speciosa* (*MS*), an evergreen shrub native to the Indian subcontinent, has garnered increasing attention for its remarkable nutritional profile and wide-ranging bioactivities. It is primarily cultivated in tropical and subtropical regions, including India, Vietnam, Thailand, and particularly southern China, where it is commonly grown in provinces such as Guangxi, Guangdong, and Yunnan [1,2,3]. In these areas, MS is traditionally used for nutritional supplementation and the management of various ailments, especially in rural and low-income communities [2,3].

In recent years, the commercial cultivation of MS has expanded due to its potential applications in the food, pharmaceutical, and agricultural industries, contributing to local economies through both domestic use and export potential [2]. From a sustainability perspective, MS demonstrates favorable agronomic traits, including tolerance to poor soil conditions, drought resistance, and minimal reliance on synthetic agrochemicals. These characteristics make it suitable for low-input agriculture and cultivation on marginal lands [2,3]. However, the growing commercial demand has led to concerns regarding land use efficiency, biodiversity impacts, and harvesting intensity in certain regions [2,3]. Long-term ecological monitoring and the implementation of sustainable cultivation practices are therefore essential to ensure that MS production remains environmentally and economically viable [3,4].

MS and its by-products serve as valuable sources of essential macronutrients, including proteins and carbohydrates, as well as micronutrients like minerals [4]. In addition, they contain a diverse array of bioactive phytochemicals such as flavonoids, saponins, and phytosterols, which contribute to their functional and therapeutic potential [4]. These compounds contribute to the health-promoting properties of MS, forming a solid basis for its application in functional foods and pharmaceuticals [5,6]. MS and its by-products have shown notable biological activities such as antioxidant, antimicrobial, anti-inflammatory, and anticancer effects [7,8,9]. These bioactive components promote overall health through various mechanisms, including immune modulation, lipid metabolism regulation, gut health improvement, and depression alleviation [1,10]. Furthermore, the potential of MS and its by-products in preventing and treating chronic diseases has garnered growing interest, particularly in functional food research.

Although recent studies have significantly advanced our understanding of the nutritional composition and bioactive properties of MS and its by-products, current knowledge remains scattered, and an integrated evaluation of their pharmacological effects, safety profiles, and industrial applications is still lacking. Most existing reviews focus on isolated biological functions or specific constituents, without comprehensively covering both the functional attributes and practical utilization of MS across multiple industries. Compared to previous literature, this review provides a broader and more integrative perspective by highlighting not only the nutritional and phytochemical components but also summarizing emerging evidence on safety and toxicity, which has rarely been addressed in earlier reviews. In addition, the current applications of MS and its by-products in the food, agriculture, pharmaceutical, and cosmetic sectors are reviewed to enhance understanding of their practical uses. Finally, we identify key research gaps and propose directions for future studies to promote the safe and sustainable development of MS and its derivatives.

## 2. Nutritional Components of MS

As outlined in Table 1, the primary nutritional components of MS and its by-products include proteins, fatty acids, carbohydrates, vitamins, and minerals, all of which contribute to their diverse applications.

### 2.1. Protein

**Figure 1:**
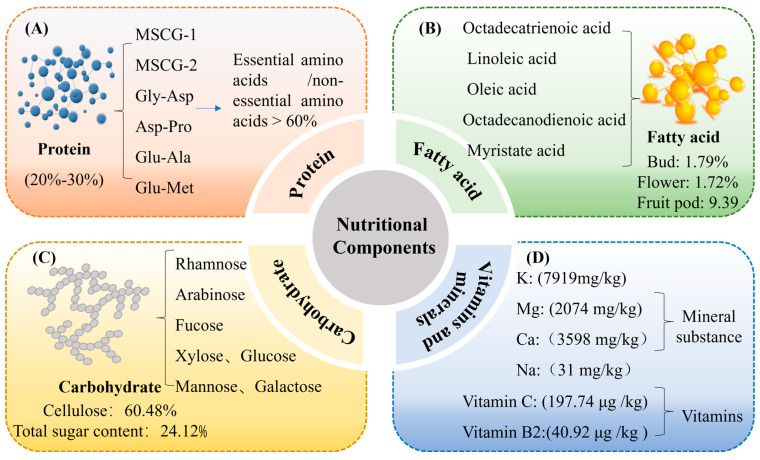
Nutritional compositions of MS and its by-products: Protein (**A**) (Gly: Glycine; Asp: Aspartic acid; Pro: Proline; Glu: Glutamic acid; Ala: Alanine; Met: Methionine); Fatty acid (**B**); Carbohydrates (**C**); Vitamins and Minerals (**D**).

Proteins and amino acids represent major nutritional constituents of MS and its by-products, underpinning its potential as a high-quality plant-based protein source. The total protein content in MS ranges from 20% to 30% of dry weight, with leaves containing up to 20% protein [11]. This relatively high protein level highlights the nutritional potential of MS; however, the protein quality, including amino acid composition and digestibility, requires further comparative evaluation against conventional leguminous crops to fully establish its suitability for human consumption. The amino acid composition of MS notably exceeds the FAO/WHO recommended reference pattern, particularly in terms of essential amino acid abundance and balance, thereby supporting its role in promoting metabolic health and maintaining nutritional homeostasis [1,12]. Beyond quantity, the bioactivity of MS proteins contributes to its functional food potential. For instance, glycoproteins such as MS seed crude glycoprotein-1 and MS seed crude glycoprotein-2, isolated from MS seeds, have been shown to possess immunomodulatory and antioxidative properties [13], which are closely linked to their specific glycosylation patterns and tertiary structures. Additionally, MS-derived peptides, including the dipeptides glycine–aspartic acid (Gly-Asp) and aspartic acid–proline (Asp-Pro), show notable free radical-scavenging and angiotensin I-converting enzyme (ACE)-inhibitory activities (Figure 1A). Reflecting their structural motifs that facilitate interaction with biological targets [11].

Efficient extraction of MS proteins typically involves aqueous or enzymatic extraction methods, sometimes coupled with membrane separation or ultrafiltration, to preserve functional integrity and yield bioactive fractions [11,14,15]. Recent advances in green extraction technologies—such as ultrasound-assisted and subcritical water extraction—have shown promise in enhancing extraction efficiency while maintaining the native structure of bioactive peptides [14,15]. The structure–activity relationship SAR studies further underscore that specific sequence motifs, hydrophobicity, and molecular conformation significantly influence the bioactivity of MS-derived peptides, guiding the future development of peptide-based nutraceuticals [11,15].

**Table 1:** Nutritional compositions of MS and its by-products.

Classifications	Extraction Method	Nutrition Compositions	Parts	Contents	References
Amino acid	Weighed 2 g of sample powder was mixed with 10 mL of 6 M hydrochloric acid, sealed, and hydrolyzed at 110 °C for 22 h	Aspartic acid	Seeds	1.21 g/100 g	[1]
		Leucine	Seeds	0.83 g/100 g	
		Lysine	Seeds	0.82 g/100 g	
		Serine	Seeds	0.71 g/100 g	
		Glutamic acid	Seeds	1.76 g/100 g	
		Proline	Seeds	0.58 g/100 g	
		Alanine	Seeds	0.62 g/100 g	
		Isoleucine	Seeds	0.38 g/100 g	
		Histidine	Seeds	0.30 g/100 g	
		Tyrosine	Seeds	0.23 g/100 g	
		Methionine	Seeds	0.05 g/100 g	
		Phenylalanine	Seeds	0.61 g/100 g	
		Threonine	Seeds	0.64 g/100 g	
		Valine	Seeds	0.57 g/100 g	
		Arginine	Seeds	0.48 g/100 g	
		Glycine	Seeds	0.52 g/100 g	
	Weighed 2 g of sample powder was mixed with 10 mL of 6 M hydrochloric acid, sealed, and hydrolyzed at 110 °C for 22 h	Aspartic acid	Leaves	1.57 g/100 g	[1]
		Glutamic acid	Leaves	1.73 g/100 g	
		Proline	Leaves	1.57 g/100 g	
		Alanine	Leaves	0.99 g/100 g	
		Leucine	Leaves	1.20 g/100 g	
		Lysine	Leaves	1.10 g/100 g	
		Serine	Leaves	0.70 g/100 g	
		Threonine	Leaves	0.76 g/100 g	
		Arginine	Leaves	0.77 g/100 g	
		Glycine	Leaves	0.81 g/100 g	
		Isoleucine	Leaves	0.65 g/100 g	
		Phenylalanine	Leaves	0.71 g/100 g	
		Histidine	Leaves	0.36 g/100 g	
		Tyrosine	Leaves	0.28 g/100 g	
		Methionine	Leaves	0.02 g/100 g	
		Valine	Leaves	0.85 g/100 g	
	Weighed 2 g of sample powder was mixed with 10 mL of 6 M hydrochloric acid, sealed, and hydrolyzed at 110 °C for 22 h	Aspartic acid	Flower	1.79 g/100 g	[1]
		Glutamic acid	Flower	1.01 g/100 g	
		Proline	Flower	0.79 g/100 g	
		Alanine	Flower	0.69 g/100 g	
		Leucine	Flower	0.63 g/100 g	
		Lysine	Flower	0.60 g/100 g	
		Serine	Flower	0.55 g/100 g	
		Threonine	Flower	0.52 g/100 g	
		Valine	Flower	0.52 g/100 g	
		Arginine	Flower	0.49 g/100 g	
		Glycine	Flower	0.41 g/100 g	
		Isoleucine	Flower	0.37 g/100 g	
		Histidine	Flower	0.27 g/100 g	
		Tyrosine	Flower	0.14 g/100 g	
		Phenylalanine	Flower	0.35 g/100 g	
	Two grams of sample powder were weighed and mixed with 10 mL of deionized water, then ground in a mortar for 10 min. Subsequently, 20 mL of 10% sulfosalicylic acid was added, and the mixture was transferred to a 50 mL centrifuge tube. The tube was stored at 4 °C for 17 h.	Aspartic acid	Champ	0.0033 g/100 g	[12]
		Glutamic acid	Champ	0.0071 g/100 g	
		Proline	Champ	0.0472 g/100 g	
		Alanine	Champ	0.0064 g/100 g	
		Leucine	Champ	0.0026 g/100 g	
		Lysine	Champ	0.0013 g/100 g	
		Serine	Champ	0.1141 g/100 g	
		Threonine	Champ	0.0089 g/100 g	
		Glycine	Champ	0.0071 g/100 g	
		Valine	Champ	0.0026 g/100 g	
		Arginine	Champ	0.0201 g/100 g	
		Tyrosine	Champ	0.1660 g/100 g	
		Phenylalanine	Champ	0.0011 g/100 g	
		Histidine	Champ	0.0054 g/100 g	
Fatty acid	Weighed 10.0 g of seeds, mixed with 100 mg of gallic acid, and added 95% ethanol and HCl for hydrolysis at 40 min. After cooling, extracted with ether-petroleum ether, and concentrated under reduced pressure to obtain the fat extract. Refluxed with NaOH-methanol, followed by BF3-methanol reflux. Extracted and processed with n-heptane, and finally dried with anhydrous sodium sulfate.	Lauric acid	Seeds	0.0156%	[1]
		Myristic acid	Seeds	0.130%	
		Pentadecanoic acid	Seeds	0.0121%	
		Docosanoic acid	Seeds	1.16%	
		Palmitic acid	Seeds	21.3%	
		Palmitoleic acid	Seeds	0.338%	
		Heptadecanoic acid	Seeds	0.127%	
		Erucic acid	Seeds	0.0811%	
		Stearic acid	Seeds	4.99%	
		Oleic acid	Seeds	27.3%	
		Linoleic acid	Seeds	41.9%	
		Linolenic acid	Seeds	0.807%	
		Eicosanoenoic acid	Seeds	0.305%	
		Heneicosanoic acid	Seeds	0.0559%	
		Eicosadienoic acid	Seeds	0.0230%	
		Arachidic acid	Seeds	0.580%	
Monosaccharide	Hydrolyzed the polysaccharides using TFA (2 M) at 105 °C, followed by methanol distillation to remove excess TFA. Finally, diluted the hydrolysate with distilled water and measured it by ion chromatography.	Rhamnose	Champ	-	[14]
		Arabinose	Champ		
		Fucose	Champ		
		Glucose	Champ		
		Mannose	Champ		
		Fructose	Champ		
		Galactose	Champ		
Mineral substance	Weighed 0.20 g of the sample and performed digestion with nitric acid (HNO_3_). The sample was ventilated at 160 °C for 60 min, and the residue was then diluted with water to a final volume of 50 mL.	Ni	Seeds	3.0 mg/kg	[1]
		Cu	Seeds	7.1 mg/kg	
		Na	Seeds	<3 mg/kg	
		Zn	Seeds	38 mg/kg	
		Mg	Seeds	2074 mg/kg	
		Sb	Seeds	<0.1 mg/kg	
		Pb	Seeds	<0.1 mg/kg	
		Al	Seeds	<2 mg/kg	
		Se	Seeds	0.59 mg/kg	
		K	Seeds	7919 mg/kg	
		Rb	Seeds	18 mg/kg	
		Ca	Seeds	925 mg/kg	
		Sr	Seeds	0.24 mg/kg	
		Ti	Seeds	<0.1 mg/kg	
		Ag	Seeds	<0.1 mg/kg	
		V	Seeds	<0.1 mg/kg	
		Cd	Seeds	<0.05 mg/kg	
		Cr	Seeds	<0.1 mg/kg	
		Sn	Seeds	<0.1 mg/kg	
		Mn	Seeds	37 mg/kg	
		Fe	Seeds	34 mg/kg	
		Hg	Seeds	<0.05 mg/kg	
		Ba	Seeds	0.42 mg/kg	
		Mo	Seeds	0.26 mg/kg	
		Li	Seeds	<0.1 mg/kg	
		B	Seeds	7.8 mg/kg	
	Weighed 0.20 g of the sample and performed digestion with nitric acid (HNO_3_). The sample was ventilated at 160 °C for 60 min, and the residue was then diluted with water to a final volume of 50 mL.	Ni	Leaves	0.91 mg/kg	[1]
		Cu	Leaves	5.7 mg/kg	
		Na	Leaves	31 mg/kg	
		Zn	Leaves	26 mg/kg	
		Mg	Leaves	1417 mg/kg	
		Al	Leaves	54 mg/kg	
		Se	Leaves	0.24 mg/kg	
		K	Leaves	9547 mg/kg	
		Rb	Leaves	20 mg/kg	
		Ca	Leaves	3598 mg/kg	
		Sr	Leaves	6.2 mg/kg	
		Ti	Leaves	0.44 mg/kg	
		Ag	Leaves	<0.1 mg/kg	
		V	Leaves	<0.1 mg/kg	
		Cd	Leaves	<0.05 mg/kg	
		Cr	Leaves	0.52 mg/kg	
		Sn	Leaves	<0.1 mg/kg	
		Mn	Leaves	137 mg/kg	
		Sb	Leaves	<0.1 mg/kg	
		Fe	Leaves	106 mg/kg	
		Hg	Leaves	<0.05 mg/kg	
		Ba	Leaves	8.4 mg/kg	
		Mo	Leaves	<0.1 mg/kg	
		Li	Leaves	<0.1 mg/kg	
		B	Leaves	16 mg/kg	
		Pb	Leaves	0.47 mg/kg	
		Ni	Flower	2.2 mg/kg	[1]
		Cu	Flower	5.3 mg/kg	
		Na	Flower	22 mg/kg	
		Zn	Flower	19 mg/kg	
		Mg	Flower	1101 mg/kg	
		Al	Flower	31 mg/kg	
		Se	Flower	<0.1 mg/kg	
		K	Flower	10,642 mg/kg	
		Rb	Flower	24 mg/kg	
		Ca	Flower	1436 mg/kg	
		Sr	Flower	2.2 mg/kg	
		Ti	Flower	0.44 mg/kg	
		Ag	Flower	<0.1 mg/kg	
		V	Flower	<0.1 mg/kg	
		Cd	Flower	<0.05 mg/kg	
		Cr	Flower	0.42 mg/kg	
		Sn	Flower	<0.1 mg/kg	
		Mn	Flower	86 mg/kg	
		Sb	Flower	<0.1 mg/kg	
		Fe	Flower	78 mg/kg	
		Mo	Flower	0.37 mg/kg	
		Li	Flower	<0.1 mg/kg	
		B	Flower	8.3 mg/kg	
		Pb	Flower	0.48 mg/kg	
		Ba	Flower	4.3 mg/kg	
		Hg	Flower	<0.05 mg/kg	
	Weighed 0.5 g of MS powder into a 50 mL PTFE digestion vessel, added 8 mL of concentrated nitric acid and 2 mL of 30% hydrogen peroxide solution, and performed heating digestion for 30 min	B	Champ	5.2 mg/kg	[12]
		Mg	Champ	2206.40 mg/kg	
		Al	Champ	42.73 mg/kg	
		Ca	Champ	2878.20 mg/kg	
		V	Champ	0.14 mg/kg	
		Mn	Champ	18.72 mg/kg	
		Cr	Champ	0.16 mg/kg	
		Rh	Champ	0.01 mg/kg	
		Cu	Champ	5.97 mg/kg	
		Fe	Champ	118.85 mg/kg	
		Co	Champ	0.17 mg/kg	
		Ni	Champ	3.02 mg/kg	
		Sr	Champ	56.93 mg/kg	
		Zn	Champ	13.27 mg/kg	
		Se	Champ	0.03 mg/kg	
		Mo	Champ	0.86 mg/kg	
		Re	Champ	0.01 mg/kg	
		Cd	Champ	0.06 mg/kg	
		As	Champ	0.03 mg/kg	
		Hg	Champ	0.11 mg/kg	
		Pb	Champ	1.29 mg/kg	

### 2.2. Fatty Acids

As shown in Figure 1B, MS seeds contain the highest levels of fatty acids among various plant tissues. The major fatty acids identified include linoleic acid (C18:2, 41.9%), oleic acid (C18:1, 27.3%), palmitic acid (C16:0, 21.3%), stearic acid (C18:0, 4.99%), and behenic acid (C22:0, 1.16%) [1]. Among these, linoleic and oleic acids are well known for their physiological benefits, including anti-inflammatory, antioxidant, and lipid-lowering effects, which contribute to cardiovascular protection and metabolic health [1].

In addition to seeds, other parts of MS, such as flower buds, flowers, and fruit pods, also exhibit notable fatty acid content. For example, fruit pods have been reported to contain approximately 12.5% total fatty acids, with linoleic and oleic acids as the predominant components [16]. The observed variation in fatty acid composition across different tissues may be attributed to developmental stage, metabolic specialization, and environmental adaptation.

### 2.3. Carbohydrates

Carbohydrates, especially bioactive polysaccharides, constitute a vital functional component of MS Champ, contributing not only to its nutritional profile but also to its therapeutic potential. As shown in Figure 1C, dried MS Champ contains a high proportion of insoluble dietary fiber, including cellulose (up to 60.48%), and total sugars (24.12%), with heteropolysaccharides as the major bioactive constituents [14]. Structural analysis has revealed that these polysaccharides are composed of monosaccharide units such as rhamnose, arabinose, fucose, xylose, mannose, glucose, and galactose, arranged in complex branching patterns that influence their physiological activity.

Recent studies have shown that MS Champ-derived polysaccharides exert immunomodulatory and gut-protective effects. For instance, Huang et al. (2020) [17] reported that these polysaccharides enhance the phagocytic activity of macrophages, promote the secretion of cytokines such as interleukin-6 (IL-6) and tumor necrosis factor-alpha (TNF-α), and stimulate splenic lymphocyte proliferation. Furthermore, they have demonstrated protective effects against cyclophosphamide-induced immunosuppression and intestinal mucosal damage by restoring intestinal barrier function and modulating gut microbiota composition [18]. These findings suggest that MS polysaccharides hold promise as prebiotic agents and immune-supporting ingredients in functional food and nutraceutical applications.

### 2.4. Vitamins and Minerals

MS Champ contains essential vitamins, primarily vitamin C and vitamin B2, both of which are crucial components of its nutritional profile. Vitamin C content in MS Champ is significantly influenced by regional and climatic factors. For example, MS Champ from Hainan exhibits markedly higher vitamin C levels compared to those from Guangxi, likely due to variations in soil composition, climate, and growing conditions [12]. In contrast, the vitamin B2 content remains relatively stable across different regions, indicating that its levels are less affected by environmental conditions [12,19]

In addition to vitamins, MS and its by-products are rich in several essential minerals, including potassium, magnesium, and selenium (Figure 1D). The potassium and magnesium content in MS seeds is 2074 mg/kg and 7919 mg/kg, respectively, while the magnesium content in the leaves can reach up to 9547 mg/kg [1]. Notably, selenium content in MS is 0.59 mg/kg, which is significantly higher than that found in many conventional foods [1].

## 3. Chemical Compositions of MS and By-Products

### 3.1. Flavonoid Compounds

**Figure 2:**
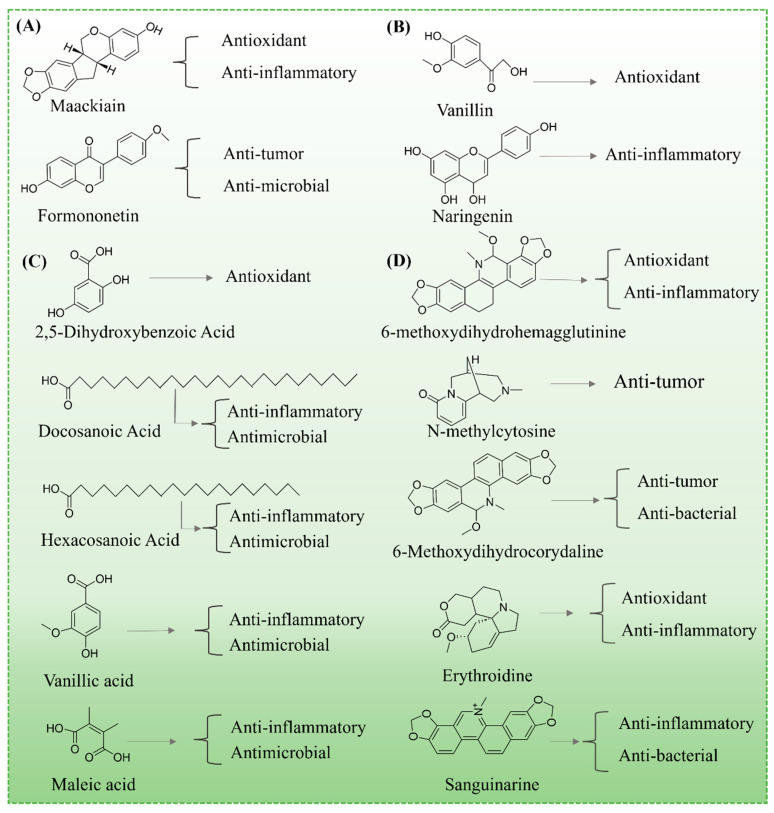
Chemical compositions and activity of MS and its by-products: Flavonoid Compounds (**A**); Phenolic Acids and Derivatives (**B**); Organic Acids (**C**); Alkaloids (**D**).

Flavonoids are among the most abundant and pharmacologically active secondary metabolites in MS Champ. Targeted phytochemical analyses have identified 35 distinct flavonoids in MS Champ (Table 2), with maackiain and formononetin as the major bioactive constituents [4,6]. As illustrated in Figure 2A, Maackiain has demonstrated strong antioxidant and anti-inflammatory properties, primarily through the inhibition of ROS generation and downregulation of pro-inflammatory cytokines, offering protective effects against cardiovascular and metabolic disorders [20]. Formononetin, on the other hand, exhibits notable anticancer potential by promoting apoptosis and suppressing proliferation in breast and lung cancer models [21]. These compounds represent key functional components contributing to the therapeutic potential of MS, warranting further investigation into their mechanisms of action and potential applications in functional food and pharmaceutical formulations.

### 3.2. Phenolic Acids and Derivatives

Phenolic acids, consisting of a benzene ring bonded to hydroxyl and carboxyl groups, are vital functional constituents in MS and its by-products. Seventeen phenolic acids and their derivatives have been identified in MS and by-products (Table 2), including vanillic acid and dihydroquercetin [21]. As illustrated in Figure 2B, vanillic acid is particularly known for its exceptional antioxidant activity. It protects cells by scavenging free radicals, inhibiting lipid peroxidation, and reducing oxidative stress, which in turn lowers the risk of chronic diseases such as cardiovascular diseases, diabetes, and neurodegenerative disorders [22]. Mechanistically, vanillic acid has been shown to modulate key antioxidant enzymes such as superoxide dismutase and catalase, providing cellular protection [23].

Dihydroquercetin, a potent flavonoid compound, exhibits prominent anti-inflammatory properties by regulating inflammatory signaling pathways, including the nuclear factor kappa B (NF-κB) and mitogen-activated protein kinase (MAPK) pathways, and inhibiting the release of pro-inflammatory cytokines such as TNF-α and IL-6 [5,24]. These effects not only alleviate inflammation but also contribute to the management of diseases related to chronic inflammation, such as arthritis and inflammatory bowel disease.

### 3.3. Triterpenoid Compounds

Triterpenoid compounds, which consist of triterpenes or steroid molecules glycosidically linked to sugar moieties, are known for their remarkable anti-inflammatory, anticancer, and immune-modulatory activities. These bioactive compounds have become key targets in pharmacological research and the development of health supplements. Recent studies have identified 38 triterpenoid compounds in MS Champ extracts (Table 2), with the main aglycones being oleanolic acid and ursolic acid [25]. Oleanolic acid has been shown to exert potent anti-inflammatory and antioxidant effects by inhibiting the expression of pro-inflammatory factors and reducing oxidative stress [26]. In contrast, ursolic acid demonstrates anticancer potential by regulating the cell cycle, inducing apoptosis, and inhibiting tumor cell proliferation and metastasis [27]. Additionally, the sugar components in MS Champ saponins, primarily d-glucose and d-glucuronic acid, enhance the solubility and bioavailability of saponins, thereby amplifying their biological effects. These functional properties underscore the potential of triterpenoid compounds in drug development, functional foods, and dietary supplements.

### 3.4. Organic Acids

Organic acids are widely distributed in plants, playing essential roles in regulating growth, enhancing stress resistance, and improving disease resilience [28]. Due to their significant bioactivity, these compounds have become a focal point in pharmacological research and the development of functional products. Several organic acids have been identified in MS Champ (Table 2), including 2,5-dihydroxybenzoic acid, behenic acid, maleic acid, and vanillic acid [4]. As illustrated in Figure 2C, 2,5-dihydroxybenzoic acid exhibits remarkable antioxidant activity, scavenging free radicals and inhibiting lipid peroxidation, thereby reducing cellular oxidative damage [29]. Maleic acid and vanillic acid are particularly known for their antimicrobial and anti-inflammatory effects, effectively modulating inflammatory responses and inhibiting pathogen growth [29,30]. The diversity and pronounced bioactivity of organic acids in MS Champ underscore their potential as valuable resources for the development of functional foods, pharmaceuticals, and health supplements.

### 3.5. Phytosterols

Phytosterols, a class of naturally occurring compounds, have attracted significant attention due to their potent biological activities, particularly their lipid-lowering, antioxidant, anti-inflammatory, immune-modulatory, and anticancer effects. MS and its by-products are rich in a variety of sterol compounds, including β-sitosterol, γ-sitosterol, dandelion sterol, and δ-5-ergosterol [31,32]. Among these, β-sitosterol has been shown to effectively lower total cholesterol levels by inhibiting intestinal cholesterol absorption and promoting bile acid excretion, thus reducing the risk of cardiovascular diseases [33]. γ-Sitosterol has demonstrated immune-regulatory properties, enhancing immune responses and strengthening pathogen defense [34]. Furthermore, dandelion sterol exhibits notable antidiabetic potential. The diverse physiological functions of phytosterols in MS highlight their promising potential for development in functional foods, pharmaceuticals, and health products.

### 3.6. Alkaloids

Alkaloids, nitrogen-containing natural compounds, have garnered considerable attention due to their broad spectrum of biological activities, including antibacterial, antiviral, analgesic, anticancer, and neuroregulatory effects. Several alkaloid constituents have been identified in MS, notably 6-methoxydihydrohemagglutinine and sanguinarine [4]. As shown in Figure 2D, 6-methoxydihydrohemagglutinine effectively inhibits the growth of various bacteria and fungi, significantly reducing the risk of infectious diseases [4]. Sanguinarine is particularly recognized for its anticancer properties, inducing apoptosis and inhibiting the proliferation and metastasis of cancer cells, thus suppressing tumor growth [35]. The diverse biological activities of these alkaloids underscore the therapeutic potential of MS in antibacterial, anti-inflammatory, analgesic, and anticancer applications.

### 3.7. Esters

Esters, key secondary metabolites in plants, have garnered significant attention from both academic and industrial sectors due to their diverse biological activities. Studies have shown that lipid-soluble extracts from MS contain 34 ester compounds (Table 2), including methyl coumarate and linoleic acid ethyl ester [6,36]. Methyl coumarate is known for its ability to reduce oxidative damage by scavenging free radicals and inhibiting lipid peroxidation, thereby significantly lowering the risk of chronic diseases [37]. Linoleic acid ethyl ester is particularly effective in anti-inflammatory and anticancer applications, as it suppresses tumor cell proliferation, modulates inflammatory responses, and alleviates inflammation, ultimately inhibiting tumor growth [38]. These distinct biological activities of ester compounds offer promising opportunities for the development of MS in natural medicine, health supplements, and functional foods.

### 3.8. Other Compounds

In addition to the aforementioned components, MS contains a rich array of phenylpropanoid and terpenoid compounds, which are of considerable interest due to their diverse biological activities. The phenylpropanoids in MS include dihydro-dehydrodiconiferyl alcohol, taxol, and psoralen [4]. Both dihydro-dehydrodiconiferyl alcohol and taxol are particularly valued for their potent antioxidant properties, which reduce oxidative stress and protect cells from oxidative damage [39,40]. Psoralen has shown promising anticancer effects, significantly inhibiting tumor cell proliferation and inducing apoptosis [41,42,43]. The terpenoid compounds in MS, including phytol, also play vital roles in antibacterial activity, immune modulation, and enhancement of metabolic functions. Overall, the phenylpropanoids and terpenoids in MS provide a valuable foundation for applications in antioxidation, anti-inflammation, anticancer, and other therapeutic domains, offering a promising basis for future drug discovery and functional food innovation.

**Table 2:** Chemical compositions of MS and its by-products.

Classify	Plant Parts	Names	Extraction Solvents	Analysis Methods	References
Flavonoids	Champ	Naringenin	95% ethanol	Silica gel column chromatography Sephadex LH-20 chromatography ODS column chromatography	[6]
	Champ	Garbanzo			
	Champ	7-hydroxy-6,4- dimethoxy isoflavone			
	Champ	2,5,7-trihydroxy-4-methoxy isoflavone			
	Champ	2-hydroxy biochanin A			
	Champ	6-methoxycalopogonium isoflavone A			
	Champ	4,4, -dihydroxy-2′-methoxy chalcone			
	Champ	2,4-dihydroxy-4-methoxy chalcone			
	Champ	2,4,4, α-tetrahydroxy dihydrogen chalcone			
	Champ	3,4-dihydroxy-7-methoxy isoflavones			
	Champ	4-hydroxy-2,4-dimethoxy chalcone			
	Champ	2,4, a-trihydroxy-4-methoxy-dihydrogen chalcone			
	Champ	3,4,7-trihydroxy isoflavones			
	Champ	Formononetin	95% ethanol	Silica gel column chromatography Sephadex LH-20 chromatography ODS column chromatography	[6]
	Champ	3,4,2′,4′-tetrahydroxychalcone			
	Champ	Homopterocarpin	Ethanol Ethyl acetate Petroleum ether	HPLC-MS	[32]
	Champ	Isoliquiritigenin			
	Champ	Maackiain			
	Champ	Pterocarpan			
	Champ	Medicarpin	95% ethanol	Silica gel column chromatography Sephadex LH-20 chromatography ODS column chromatography	[44]
	Champ	3,7-dihydroxy-2,4′-dimethoxy isoflavone			
	Champ	4,2,4-trihydroxy chalcone			
	Champ	4-hydroxy-7-methoxy flavanone			
	Champ	Bisdemethoxycurcumin	95% ethanol	Silica gel column chromatography Sephadex LH-20 chromatography ODS column chromatography	[45]
	Champ	Corylifolin			
	Champ	Quercetin			
	Champ	Isoquercitrin			
	Champ	Licochalcone A			
	Champ	Tectorigenin			
	Champ	Liquiritigenin			
	Champ	Sulfuretin			
	Champ	Amentoflavone			
Phenolic acid and derivatives	Champ	1,4-butanediol	70% methanol,	UHPLC-MS/MS	[8]
	Champ	Isotrifoliol			
	Champ	2-pentadecanone			
	Champ	3-hydroxy-4-methoxy benzoic acid			
	Champ	Vanillic acid			
	Champ	Dicoumarol			
	Champ	Syringin			
	Champ	(+/−)-Gingerol			
	Champ	Nanillic acid			
	Champ	Secoisolariciresinol			
	Champ	Leiocarposide			
Triterenoids	Champ	3β-olean-12-en-28,29-dioic acid 3-O-α-l-arabinopyranosyl-(1 → 2)-α-l-rhamnopyranosyl-(1 → 2)-[α-l-arabinopyranosyl-(1 → 3)]-β-d-galactopyranoside	70% methanol,	UHPLC-MS/MS	[21]
	Champ	3β,22,24-trihydroxyolean-12-en-29-oic acid 3-O-α-l-rhamnopyranosyl-(1 → 2)-α-l-rhamnopyranosyl-(1 → 2)-β-d-glucuronopyranoyl-22-O-β-d-glucopyranoside			
	Champ	Betulinic acid 3-O-β-d-glucopyranosyl-(1 → 6)-β-d-glucopyranosyl-(1 → 6)-β-d-glucopyranosyl-28-O-β-d-glucopyranoside			
	Champ	Saikogenin G 3-O-β-d-glucopyranosyl-(1 → 6)-β-d-glucopyranosyl-(1 → 6)-β-d-glucopyranoside			
	Champ	3α-hydroxy-11-oxoolean-12-en-30-oic acid 3-O-α-l-rhamnopyranosyl-(1 → 2)-β-d-glucuronopyranoside			
	Champ	Betulinic acid 3-O-α-l-arabinopyranosyl-(1 → 2)-α-l-rhamnopyranosyl-(1 → 2)-β-D-galactopyranoside			
	Champ	Soyasapogrnol B 3-O-α-l-arabinopyranosyl-(1 → 2)-β-d-galactopyranosyl-(1 → 2)- glucuronopyranosyl-22- O-β-d-glucopyranoside			
	Champ	3β,22,24-trihydroxyolean-12-en-29-oic acid 3-O-α-l-rhamnopyranosyl-(1 → 2)-α-l-rhamnopyranosyl-(1 → 2)-β-d-glucuronopyranoyl-22-O-β-d-glucopyranoside			
	Champ	Saikogenin G 3-O-α-l-arabinopyranosyl-(1 → 2)-α-l-rhamnopyranosyl-(1 → 2)-[α-l-arabinopyranosyl-(1 → 3)]-β-d-galactopyranoside			
	Champ	3β-olean-12-en-28,29-dioic acid 3-O-α-l-arabinopyranosyl-(1 → 2)-α-l-rhamnopyranosyl-(1 → 2)-β-d-galactopyranoside			
	Champ	Betulinic acid 3-O-α-l-arabinopyranosyl-(1 → 2)-β-d-galactopyranosyl-(1 → 2)-glucuronopyranosyl-28-O-β-d-glucopyranoside			
	Champ	3β,22,24-trihydroxyolean-12-en-29-oic acid 3-O-α-l-rhamnopyranosyl-(1 → 2)-β-d-galactopyranosyl-(1 → 2)-glucuronopyranosyl-22-O-β-d-glucopyranoside			
	Champ	3β,22,24-trihydroxyolean-12-en-29-oic acid 3-O-α-l-rhamnopyranosyl-(1 → 2)-α-l-rhamnopyranosyl-(1 → 2)-β-d-glucuronopyranoyl-22-O-β-d-glucopyranoside			
	Champ	Oleanolic acid 3-O-α-l-rhamnopyranosyl-(1 → 2)-β-d-galactopyranosyl-(1 → 2)-glucuronopyranosyl-28- O-β-d-glucopyranoside			
	Champ	3α-hydroxy-11-oxoolean-12-en-30-oic acid 3-O-α-l-rhamnopyranosyl-(1 → 2)-β-d-galactopyranosyl-(1 → 2)-glucuronopyranoside			
	Champ	Soyasapogrnol B 3-O-α-l-rhamnopyranosyl-(1 → 2)-β-d-galactopyranosyl-(1 → 2)-glucuronopyranoside			
	Champ	23-hydroxyl pomalic acid 3-o-α-l-rhamnopyranosyl-(1 → 4)-β-d-glucopyranosyl-(1 → 6)-β-d-galactopyranosyl-28-o-β-d-glucopyranoside			
	Champ	23-hydroxyl pomalic acid 3-O-β-d-glucopyranosyl-(1 → 6)-β-d-glucopyranosyl-28-O-β-d-glucopyranoside			
	Champ	Betulinic acid 3-O-α-l-rhamnopyranosyl-(1 → 2)-β-d-galactopyranosyl-(1 → 2)-glucuronopyranosyl-28-O-β-d-glucopyranoside			
	Champ	22β-acetyloxy-3β,24-dihydroxyolean-12-en-29-oic acid 3-O-α-l-arabinopyranosyl-(1 → 2)-α-l-rhamnopyranosyl-(1 → 2)-[α-l-arabinopyranosyl-(1 → 3)]-β-d-galactopyranoside			
	Champ	Oleanolic acid 3-O-α-l-arabinopyranosyl-(1 → 2)-β-d-galactopyranosyl-(1 → 2)-glucuronopyranoside			
	Champ	23-hydroxyl pomalic acid 3-o-α-l-rhamnopyranosyl-(1 → 4)-β-d-glucopyranosyl-(1 → 6)-β-d-galactopyranosyl-28-o-β-d-glucopyranoside			
	Champ	Oleanolic acid 3-O-β-d-glucopyranosyl-(1 → 6)-β-d-glucopyranosyl-(1 → 6)-β-d-glucopyranosyl-28-O-β-d-glucopyranoside			
	Champ	3β,22,24-trihydroxyolean-12-en-29-oic acid 3-O-α-l-rhamnopyranosyl-(1 → 2)-β-d-glucopyranosyl-(1 → 2)-β-d-glucopyranosyl-22- O-β-d-glucopyranoside			
	Champ	22β-acetyloxy-3β,24-dihydroxyolean-12-en-29-oic acid 3-O-α-l-rhamnopyranosyl-(1 → 2)-β-d-galactopyranosyl-(1 → 2)-glucuronopyranoside			
	Champ	Betulinic acid 3-O-α-l-rhamnopyranosyl-(1 → 2)-β-d-galactopyranosyl-(1 → 2)-glucuronopyranoside			
	Champ	Betulinic acid 3-O-α-l-arabinopyranosyl-(1 → 2)-α-l-rhamnopyranosyl-(1 → 2)-β-d-glucuronopyranosyl-28-O-β-d-glucopyranoside			
	Champ	Soyasapogrnol B 3-O-α-l-rhamnopyranosyl-(1 → 2)-β-d-galactopyranosyl-(1 → 2)-glucuronopyranosyl-22- O-β-d-glucopyranoside			
	Champ	3α-hydroxy-11-oxoolean-12-en-30-oic acid 3-O-α-l-rhamnopyranosyl-(1 → 4)-β-d-glucopyranosyl-(1 → 6)-β-d-galactopyranoside			
	Champ	Saikogenin G 3-O-α-l-arabinopyranosyl-(1 → 2)-α-l-rhamnopyranosyl-(1 → 2)-β-d-galactopyranoside			
	Champ	Oleanolic acid 3-O-α-l-arabinopyranosyl-(1 → 2)-α-l-rhamnopyranosyl-(1 → 2)-β-d-galactopyranoside			
	Champ	3β,22,24-trihydroxyolean-12-en-29-oic acid 3-O-α-l-rhamnopyranosyl-(1 → 2)-α-l-rhamnopyranosyl-(1 → 2)-β-d-glucuronopyranoyl-22-O-β-d-glucopyranoside			
	Champ	Saikogenin G 3-O-α-l-arabinopyranosyl-(1 → 2)-[β-d-galactopyranosyl-(1 → 3)]-β-d-galactopyranosyl-(1 → 2)-glucuronopyranoside			
	Champ	Oleanolic acid 3-O-α-l-arabinopyranosyl-(1 → 2)-α-l-rhamnopyranosyl-(1 → 2)-[α-l-arabinopyranosyl-(1 → 3)]-β-d-galactopyranoside			
	Champ	3α-hydroxy-11-oxoolean-12-en-30-oic acid 3-O-α-l-arabinopyranosyl-(1 → 4)-β-d-glucopyranosyl-(1 → 6)-β-D glucopyranoside			
	Champ	Saikogenin G 3-O-α-l-rhamnopyranosyl-(1 → 4)-β-d-glucopyranosyl-(1 → 6)-β-d-glucopyranoside			
	Champ	Betulinic acid			
Organic acids	Champ	2,5-Dihydroxybenzoic acid	95% ethanol	Silica gel column chromatography Sephadex LH-20 chromatography ODS column chromatography	[46]
	Champ	Hexacosanoic acid	Ethanol	Silica gel column chromatography Sephadex LH-20 chromatography ODS column chromatography HPLC-MS	[47]
	Champ	Dodecanoic acid			
	Champ	Nonanoic acid	95% ethanol	Silica gel column chromatography Sephadex LH-20 chromatography ODS column chromatography HPLC-MS	[48]
	Champ	Pentadecanoic acid			
	Champ	Tetradecanoic acid			
	Champ	Maleic acid			
	Champ	Vanillic acid			
	Champ	Syringic acid			
	Champ	Linoleic acid	95% ethanol	GC-MS	[49]
Esters	Champ	Caffeic acid lupeol ester	95% ethanol	GC-MS	[31]
	Champ	Capsanthin ethyl ester			
	Champ	Methyl 9,12-octadecadienoate	Ethanol	Silica gel column chromatography GC-MS	[36]
	Champ	Methyl palmitate			
	Champ	Dibutyl phthalate			
	Champ	Methyl stearate			
	Champ	Methyl oleate			
	Champ	Ethyl palmitate			
	Champ	Ethyl heptanoate			
	Champ	Methyl linoleate			
	Champ	Ethyl linoleate			
	Champ	Myristyl ethyl ester	Ethanol	GC-MS	[49]
	Champ	Avenalumic lactone			
	Champ	Linolenic acid lactone			
	Champ	Ethyl heptadecanoate			
	Champ	Ethyl stearate	Water	GC-MS	[50]
	Champ	Methyl salicylate			
	Champ	Dihydroactinidiolide			

## 4. Health-Promoting Effects of MS and By-Products

### 4.1. Antioxidant Activity

**Figure 3:**
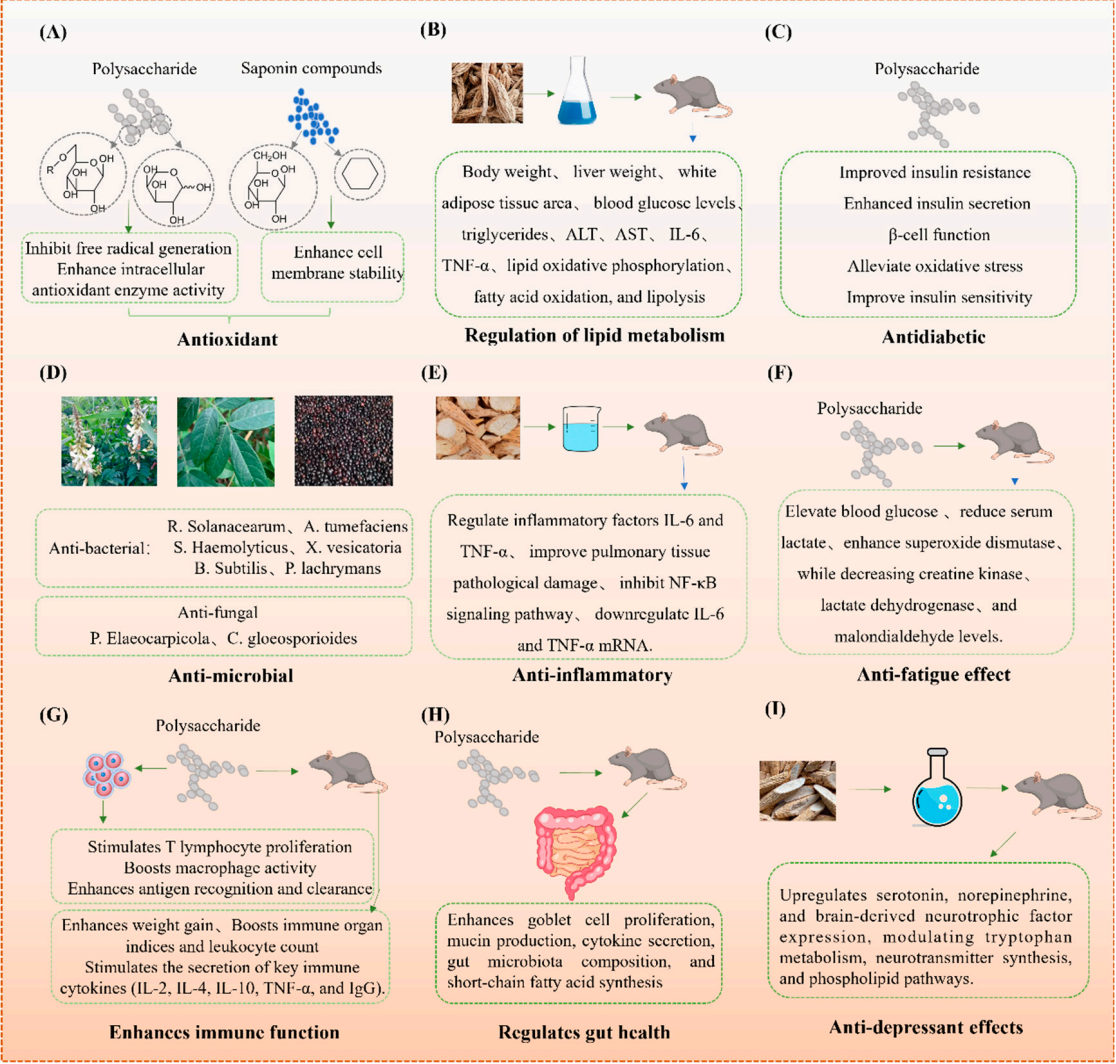
Potential biological activities of MS and its by-products: Antioxidant Activity (**A**); Regulation of Lipid Metabolism (**B**); Antidiabetic Effects (**C**); Anti-microbial (**D**); Anti-inflammatory (**E**); Antifatigue effect (**F**); Enhances immune function (**G**); Regulates gut health (**H**); Anti-depressant effects (**I**).

Recent studies have confirmed the potent antioxidant activity of MS and its by-products through both in vitro and in vivo models, mainly attributed to its polysaccharides, saponins, and flavonoids (Table 3). For instance, Bai et al. (2022) [51] reported that MS polysaccharides at a dose of 100–400 mg/kg significantly elevated superoxide dismutase (SOD) and catalase (CAT) activity in cyclophosphamide-treated mice, thereby reducing malondialdehyde (MDA) levels and oxidative tissue damage (Figure 3A). Mechanistically, these effects are associated with the upregulation of the nuclear factor erythroid 2–related factor 2/heme oxygenase-1 (Nrf2/HO-1) antioxidant signaling pathway.

Saponins extracted from MS (50–200 µg/mL) have also shown potent free radical scavenging capacity in DPPH and ABTS assays and reduced lipid peroxidation in H_2_O_2_-stressed HepG2 cells, as shown by decreased reactive oxygen species (ROS) accumulation and membrane damage [62]. Their amphipathic glycosidic-lactone structure may facilitate membrane stabilization and enhance endogenous antioxidant enzyme expression.

Flavonoids such as maackiain and formononetin exhibit strong radical scavenging activities (IC₅₀ values of 36.2 µM and 45.7 µM, respectively) and were shown to inhibit nitric oxide synthase (iNOS) and nicotinamide adenine dinucleotide phosphate (NADPH) oxidase activity in RAW264.7 macrophages under lipopolysaccharide (LPS) stimulation [63,64]. These effects are likely mediated by the inhibition of NF-κB signaling.

Interestingly, the antioxidant potential of MS extracts varies depending on the extraction solvent. Chen et al. (2021) [4] reported that aqueous extracts, rich in polysaccharides, exhibited stronger intracellular antioxidant effects in Caco-2 cells, whereas ethanol extracts, enriched in flavonoids and saponins, showed greater activity in chemical-based radical scavenging assays. These findings indicate that different bioactive fractions of MS may confer distinct antioxidant benefits depending on the biological context. However, the consistency of these effects across various in vitro and in vivo models remains limited, and further comparative studies are needed to validate their efficacy and mechanisms under physiologically relevant conditions.

### 4.2. Lipid Metabolism Regulation

Recent studies have revealed that MS regulates lipid metabolism through the synergistic action of its flavonoids and polysaccharides (Table 3). In an HFD-induced obese mouse model, administration of MS flavonoid extracts (150–300 mg/kg body weight/day for 8 weeks) led to significant reductions in body weight gain, liver weight, and epididymal white adipose tissue mass [8] (Figure 3B). In parallel, fasting blood glucose, serum Triglycerides (TG), and Total Cholesterol (TC) levels were markedly decreased.

Mechanistically, MS flavonoids were shown to activate the Adenosine Monophosphate-Activated Protein Kinase (AMPK) pathway, upregulating the expression of Carnitine Palmitoyltransferase-1 (CPT-1) and Uncoupling Protein 1 (UCP1) in brown adipose tissue, thereby promoting mitochondrial fatty acid β-oxidation and thermogenesis [65]. Moreover, phosphorylation of Hormone-Sensitive Lipase (HSL) and Adipose Triglyceride Lipase (ATGL) was enhanced, facilitating lipolysis and lipid mobilization in white adipose tissue.

MS polysaccharides (administered at 200 mg/kg in HFD mice) further improved hepatic lipid metabolism by increasing the activity of key metabolic enzymes such as LPL and ACC, while reducing serum levels of liver injury markers (ALT, AST) and inflammatory cytokines including IL-6 and TNF-α [65].

### 4.3. Antidiabetic

The antidiabetic potential of MS is mainly attributed to its bioactive polysaccharides, which are composed of glucose, arabinose, galactose, and other monosaccharides. These polysaccharides exhibit significant hypoglycemic effects in both cellular and animal models through multiple mechanisms (Figure 3C, Table 3).

In streptozotocin-induced diabetic mice, oral administration of MS polysaccharides at doses of 100–200 mg/kg body weight for 28 days resulted in a marked reduction in fasting blood glucose levels and improved glucose tolerance [66]. Mechanistically, these polysaccharides enhance insulin sensitivity by upregulating IRS-1 and PI3K, leading to increased expression of glucose transporter type 4 (GLUT4) in peripheral tissues, thereby facilitating glucose uptake [66].

Furthermore, MS polysaccharides protect pancreatic β-cells against oxidative damage by increasing the activities of antioxidant enzymes such as SOD and GSH-Px, while reducing levels of MDA and inflammatory markers (IL-6, TNF-α) [52]. These effects collectively support enhanced insulin secretion and pancreatic islet function.

Additionally, MS polysaccharides were shown to modulate the HPA axis by normalizing serum cortisol and adrenocorticotropic hormone levels, further contributing to the improvement of insulin resistance [66].

### 4.4. Antibacterial and Antiviral

The antibacterial and antiviral properties of MS are closely linked to its bioactive constituents, including phenolic acids, saponins, and polyphenols, which are unevenly distributed across different plant parts (Table 3). Notably, MS seeds and flowers possess higher concentrations of total phenolics and saponins, whereas the leaves are particularly rich in polyphenols and specific antioxidant compounds such as quercetin, kaempferol, and ascorbic acid, which contribute to their strong free radical-scavenging capacity [1].

As shown in Figure 3D, ethanol extracts from MS seed pods and flowers (100–200 mg/mL) exhibit significantly stronger antibacterial activity against both Gram-negative and Gram-positive bacteria, including *Ralstonia solanacearum*, *Agrobacterium tumefaciens*, *Staphylococcus haemolyticus*, *Xanthomonas vesicatoria*, *Bacillus subtilis*, and *Pseudomonas lachrymans*, compared to branch and leaf extracts [1]. The enhanced antibacterial efficacy is primarily attributed to the high saponin and phenolic content, which exert antimicrobial effects by increasing bacterial membrane permeability, disrupting lipid bilayers, and causing leakage of intracellular contents [25].

In addition to antibacterial effects, MS leaf extracts (150–300 μg/mL) display pronounced antifungal activity against plant pathogens such as *Phyllosticta elaeocarpicola* and *Colletotrichum gloeosporioides.* The antifungal mechanism is hypothesized to involve the induction of oxidative stress through excessive generation of ROS and inhibition of fungal cell wall synthesis, mediated by high levels of flavonoids and tannins [1].

Preliminary in vitro antiviral assessments have also shown that polyphenol-rich leaf extracts of MS inhibit the replication of influenza A virus (H1N1) and vesicular stomatitis virus (VSV) in MDCK and Vero cells, respectively, with IC_50_ values ranging from 50 to 150 μg/mL. The mechanism involves interference with viral attachment and entry, as well as downregulation of viral RNA polymerase gene expression [52].

### 4.5. Anti-Inflammatory

The anti-inflammatory activity of MS is primarily attributed to the synergistic effects of its bioactive constituents—namely, polysaccharides, flavonoids, and saponins—which collectively regulate inflammatory signaling pathways and modulate immune responses (Table 3).

Polysaccharides extracted from MS have been shown to modulate immune cell activity by enhancing macrophage phagocytosis and promoting regulatory T-cell responses, thereby maintaining immune homeostasis and reducing inflammatory overactivation [18,53]. Flavonoids, including formononetin and maackiain, exert anti-inflammatory effects by NF-κB activation and reducing the expression of iNOS and Cyclooxygenase-2 (COX-2), which are key mediators of inflammation [67]. Saponins, due to their amphiphilic nature, stabilize cell membranes and reduce the release of lysosomal enzymes and pro-inflammatory mediators, such as prostaglandins and leukotrienes [4].

As illustrated in Figure 3E, MS ethanol and aqueous extracts (at concentrations of 100–400 mg/kg body weight) significantly downregulate the expression of pro-inflammatory cytokines IL-6 and TNF-α in LPS-induced inflammation models, through inhibition of the TLR4/NF-κB and MAPK signaling pathways [53]. These molecular effects were confirmed by quantitative real-time PCR and ELISA assays, showing reduced mRNA levels and protein secretion of IL-6 and TNF-α.

In vivo experiments further substantiate the anti-inflammatory potential of MS. For example, Liu et al. (2014) [68] reported that administration of MS aqueous extracts at doses of 200 and 400 mg/kg significantly reduced ear swelling in the xylene-induced ear edema model in mice. Additionally, MS extracts effectively inhibited vascular leakage in the acetic acid-induced peritoneal capillary permeability test, indicating strong anti-exudative activity.

### 4.6. Antifatigue

The antifatigue effects of MS Champ are predominantly mediated by its polysaccharide components, which regulate energy metabolism, reduce metabolic waste accumulation, and enhance the body’s antioxidant defense system (Table 3). Polysaccharides isolated from MS have been shown to significantly alleviate physical fatigue in animal models by modulating biochemical markers associated with exercise endurance. In a murine-forced swimming test, administration of MS polysaccharides at doses of 100–400 mg/kg body weight led to a marked increase in swimming time, indicating improved endurance capacity [69]. Mechanistically, MS polysaccharides elevate hepatic and muscle glycogen stores—key energy reserves during sustained physical activity—while simultaneously increasing blood glucose levels, ensuring a continuous energy supply [4].

Moreover, MS polysaccharides reduce serum lactic acid concentration, a primary fatigue-inducing metabolite, thereby mitigating exercise-induced acidosis and delaying muscle fatigue. These effects are likely mediated through enhanced expression of lactate transporters and modulation of anaerobic glycolysis pathways [69]. Concurrently, MS polysaccharide supplementation decreases levels of fatigue biomarkers such as creatine kinase (CK) and lactate dehydrogenase (LDH), which are indicative of muscle injury and metabolic strain. In addition to modulating energy metabolism, MS polysaccharides exert antioxidant effects by increasing the activity of SOD and glutathione peroxidase (GSH-Px), while reducing MDA levels—a marker of lipid peroxidation [60]. These antioxidant actions protect skeletal muscle cells from oxidative damage and improve mitochondrial function during exercise stress.

### 4.7. Immune Enhancement

The immunoenhancing properties of MS are mainly attributed to its abundant polysaccharides and synergistic contributions from other bioactive compounds such as saponins and flavonoids (Table 3). Structurally, MS polysaccharides are composed predominantly of glucose, arabinose, and galactose residues, forming branched chains that interact with immune cells to exert regulatory effects [53].

As shown in Figure 3G, MS polysaccharides modulate both innate and adaptive immune responses through multiple mechanisms. In cellular immunity, they stimulate the proliferation of splenic T lymphocytes and promote the activation of a cluster of differentiation 4–positive (CD4^+^) and the cluster of differentiation 8–positive (CD8^+^) T cells, thereby enhancing cytotoxic responses against pathogens [70]. In humoral immunity, MS polysaccharides have been reported to increase the number of antibody-producing cells and upregulate serum immunoglobulin G levels, indicating enhanced antigen-specific responses [4].

In murine models of immunosuppression induced by cyclophosphamide, oral administration of MS polysaccharides at doses of 100–400 mg/kg significantly improved thymus and spleen indices, increased white blood cell counts, and promoted macrophage phagocytic activity [4], suggesting restoration and enhancement of both immune organ structure and function. Furthermore, MS extracts have been shown to upregulate key cytokines, including interleukins (IL-2, IL-4, IL-10) and tumor necrosis factor-alpha (TNF-α), which play essential roles in orchestrating immune signaling cascades [70]. Bioactive compounds such as saponins and flavonoids further modulate immune responses by influencing cell membrane fluidity, enhancing antigen presentation, and attenuating excessive inflammation, thereby contributing to immune homeostasis.

However, the consistency of these immunomodulatory effects across different studies remains partially constrained by variations in extraction methods, compound purity, dosages, and experimental models. While several in vivo studies using murine models report comparable trends, direct comparisons are limited due to differences in evaluation endpoints and immune challenge protocols. Therefore, further standardized and mechanistic studies, including clinical validation, are warranted to confirm the reproducibility and translational potential of MS-derived immunoregulatory compounds.

### 4.8. Gut Health Regulation

The beneficial effects of MS on gut health are primarily attributed to its polysaccharide constituents, which exert multifaceted regulatory functions on the intestinal barrier and microbial ecosystem (Table 3). As illustrated in Figure 3H, MS polysaccharides improve intestinal structure and function through both physical barrier enhancement and microbiota modulation [18].

Histological studies have shown that oral administration of MS polysaccharides at doses ranging from 100 to 400 mg/kg in mice significantly increases the number of goblet cells and the area of mucin secretion in the ileum, reinforcing the mucosal protective layer [18]. In addition, MS polysaccharides upregulate the mRNA and protein expression of key tight junction proteins, including Occludin-1, Claudin-1, and Mucin-2, thereby restoring tight junction integrity and reducing intestinal permeability, especially under chemically induced injury such as cyclophosphamide-induced mucosal damage [71].

Furthermore, MS polysaccharides exert prebiotic-like effects by altering the composition and diversity of the gut microbiota. Notably, treatment with MS polysaccharides reduces the *Firmicutes/Bacteroidetes* ratio, a known biomarker of dysbiosis, while significantly increasing the relative abundance of beneficial bacteria such as *Lactobacillaceae, Bifidobacteria*, and *Lactic Acid Bacteria* [18]. These microbial shifts are associated with increased microbial metabolic activity, particularly the enhanced production of short-chain fatty acids (SCFAs), including acetate, propionate, and butyrate [18,71].

SCFAs serve as crucial energy sources for colonocytes and play key roles in maintaining intestinal homeostasis by promoting epithelial cell regeneration, reinforcing tight junction assembly, and modulating local and systemic immune responses [72]. The increase in SCFA levels also correlates with reduced pro-inflammatory cytokine expression and improved gut immune tolerance.

### 4.9. Antidepressant

The antidepressant effects of MS are attributed to the synergistic action of its bioactive constituents, particularly L-isoleucine, decanedioic acid, and uracil, which have been identified as potential pharmacological markers (Table 3). These compounds are believed to influence central nervous system activity by modulating neurotransmitter synthesis, metabolism, and receptor signaling pathways [73].

As illustrated in Figure 3I, oral administration of MS water extracts (100–400 mg/kg/day) in murine models of depression significantly increased the levels of 5-HT, NE, and BDNF in the hippocampus and prefrontal cortex [9]. These neurochemicals are closely associated with emotional regulation, neuroplasticity, and cognitive resilience, and their upregulation corresponds with a marked reduction in depressive-like behaviors in forced swim and tail suspension tests.

Mechanistically, MS extracts appear to exert antidepressant effects through the modulation of key enzymes involved in neurotransmitter metabolism and degradation. For instance, MS has been reported to inhibit MAO-A and MAO-B, which reduces the breakdown of monoamines such as serotonin and dopamine [74]. Additionally, MS influences acetylcholine turnover by modulating AChE activity, and it alters the tryptophan-kynurenine pathway through inhibition of indoleamine 2,3-dioxygenase 1, potentially enhancing serotonin biosynthesis [9]. Furthermore, MS extract impacts phospholipid metabolism and neuronal membrane stability by regulating enzymes such as catechol-O-methyltransferase, which is involved in catecholamine degradation and is often dysregulated in patients with mood disorders [8].

## 5. Safety and Toxicological Assessment of MS

The expanding utilization of MS in the food, pharmaceutical, and dietary supplement industries has raised concerns regarding its safety and potential toxicity, prompting heightened scrutiny from both the public and regulatory agencies. Therefore, comprehensive toxicological assessments of MS extracts derived from various plant parts are crucial (Table 4).

Current research indicates that MS extracts derived from different plant components generally exhibit favorable safety profiles within defined dosage ranges. For instance, Zhao et al. (2017) [75] reported that oral administration of MS aqueous extract at a dose of 0.2 mL/10 g body weight (~2 g/kg BW) in mice did not induce observable acute toxicity, with no significant changes in clinical signs, behavior, or histopathological parameters over a 14-day observation period. Similarly, Wang et al. (2023) [1] conducted in vitro cytotoxicity assays using human fibroblasts and demonstrated that MS flower, seed, and leaf extracts at concentrations up to 200 μg/mL maintained cell viability above 90%, indicating low cytotoxic potential under physiologically relevant conditions.

Despite these encouraging findings, concerns regarding potential toxicity, particularly with prolonged high-dose exposure, persist. Chronic toxicity studies have shown that extended oral or topical use of MS does not result in significant adverse effects [76]. Research by Zhao et al. (2023) [3] revealed that continuous administration of MS champ extract over several months did not cause substantial liver or kidney damage or other toxic reactions. Consequently, MS is widely endorsed for use in food and dietary supplements due to its natural composition and low toxicity profile. However, it is important to acknowledge that the saponin content in MS may trigger adverse reactions in certain individuals, particularly in sensitive populations or under prolonged high-dose exposure. Reported effects include gastrointestinal irritation, reduced nutrient absorption, and, in rare cases, hemolytic activity, all of which warrant careful dose optimization and safety evaluation in future applications. Chronic high-dose consumption of MS has been associated with gastrointestinal disturbances, including diarrhea and discomfort [78]. The variability in these findings may stem from differences in extraction methods, dosage forms, administration routes, and experimental models. Such discrepancies underscore the need for standardized evaluation protocols and comparative toxicity assessments. Future studies should prioritize dose-response analyses, particularly across vulnerable populations, to define safe intake thresholds and minimize potential adverse effects. By addressing these inconsistencies through rigorous, harmonized toxicological investigations, the safe and effective utilization of MS and its by-products in both food and pharmaceutical applications can be better ensured.

## 6. Industrial Applications of MS and Its Products

### 6.1. Functional Beverages

**Figure 4:**
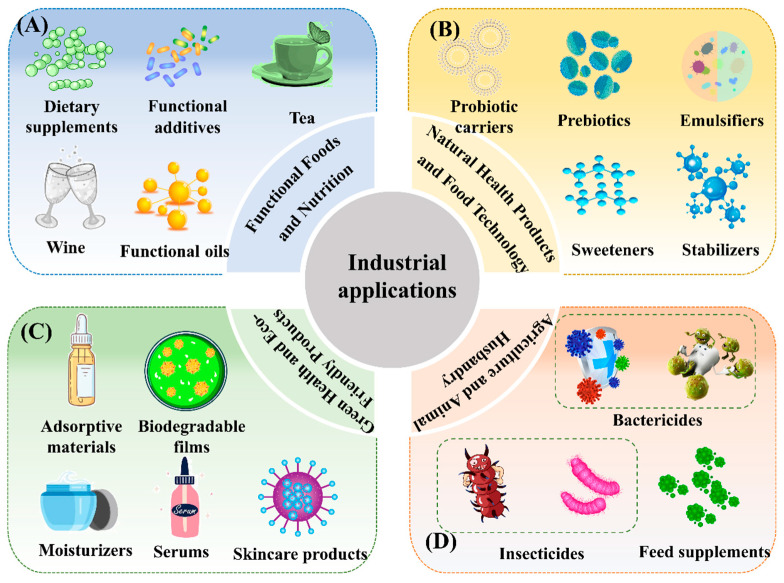
Industrial applications of MS and its products: functional foods and nutrition (**A**); natural health products and food technology (**B**); green health and Ecofriendly products (**C**); agriculture and animal husbandry (**D**).

MS tea and MS wine, as prominent functional beverages derived from MS extracts, have garnered considerable attention in the health food sector in recent years. Rich in bioactive compounds, MS tea delivers a diverse array of health benefits (Figure 4A). It exhibits strong antioxidant, anti-inflammatory, and immunomodulatory activities [17,79], while also supporting gut microbiota balance and contributing to healthy aging. Additionally, MS tea has been shown to improve digestive and metabolic functions, enhance physical performance, and promote overall vitality [58]. Its low-calorie, natural composition makes it particularly suitable for individuals managing obesity or diabetes, offering a safe and functional alternative for health maintenance [8].

MS wine, produced by infusing MS with alcohol to extract active compounds, presents unique functional properties [79]. Retaining the traditional flavor and cultural significance of alcoholic beverages, MS wine is enriched with bioactive components that support blood circulation, exhibit antioxidant properties, and alleviate fatigue [4]. Studies suggest that MS wine can enhance sleep quality, reduce mental stress, and provide auxiliary benefits for bone health and arthritis, particularly in the elderly [4]. As a functional beverage, MS wine presents a novel alternative for consumers seeking a blend of health benefits and traditional cultural elements.

### 6.2. Natural Food Emulsifiers and Stabilizers

MS polysaccharides and their acetylated derivatives have attracted significant attention as natural emulsifiers and stabilizers in the food industry. These high-molecular-weight compounds, derived from MS, exhibit excellent water solubility and viscosity-regulating properties, which enhance the texture and sensory appeal of food. As emulsifiers, MS polysaccharides stabilize oil-water emulsions by ensuring uniform dispersion and improving the emulsifying performance of food products [14]. Furthermore, these polysaccharides maintain strong stability during food processing, effectively preventing phase separation and sedimentation in stored products, thereby ensuring long-term product consistency [14].

To further optimize their emulsifying and stabilizing properties, researchers have chemically modified MS polysaccharides by introducing acetyl groups, producing acetylated derivatives [7,80]. This modification significantly enhances the hydrophobicity and emulsifying efficiency of the polysaccharides while improving their stability under acidic conditions and high temperatures [80]. Acetylated MS polysaccharides are more effective at maintaining a stable oil-water phase distribution, further improving food’s visual and organoleptic qualities [7]. Compared to traditional emulsifiers, acetylated MS polysaccharides exhibit superior performance, particularly in the production of acidic dairy beverages, emulsified sauces, and fat-based products.

### 6.3. Food and Functional Additives

In recent years, MS and its by-products have garnered significant attention as a functional food additive (Figure 4A), with its rich bioactive components playing a crucial role in the development of innovative food products. Both MS and its by-products offer valuable potential for dietary supplementation, functioning as low-calorie sweeteners while promoting gut health [71]. These compounds are particularly beneficial for the prevention and management of metabolic disorders, including diabetes and obesity [8]. Additionally, bioactive compounds derived from MS, as well as their metabolic derivatives, exhibit notable antioxidant, anti-aging, and anti-fatigue properties [10]. Their mechanisms of action include scavenging free radicals, slowing cellular aging, and modulating inflammation at the molecular level, thereby reducing the risk of chronic diseases [65,67]. The bioactive profile of MS provides a solid scientific foundation for the development of functional foods aimed at improving overall health and well-being. Compared to established cosmetic actives, MS bioactives offer added value through their multi-functionality—combining antioxidative, moisturizing, and anti-inflammatory effects in a single botanical source. Moreover, as natural, biodegradable compounds with a low allergenic profile, they align well with the growing consumer demand for clean-label and eco-conscious cosmetics.

### 6.4. Prebiotics and Probiotic Carriers

The bioactive components of MS, particularly its polysaccharides, have garnered considerable attention as potential prebiotics and carriers for probiotics (Figure 4B), emphasizing the plant’s distinct advantages in promoting gut health and enhancing functional food applications [58]. Studies indicate that MS polysaccharides, along with other soluble dietary fibers, exhibit potent prebiotic effects by stimulating the growth of beneficial bacteria, such as *Bifidobacterium* and *Lactobacillus*, which help optimize gut microbiota composition [58,71]. This modulation of the gut microbiota increases the production of SCFAs, including acetate, propionate, and butyrate, which play a critical role in enhancing gut barrier function and reducing systemic inflammation [72]. These mechanisms not only improve gut health but also show the potential to modulate immune responses and prevent metabolic disorders such as diabetes and obesity [68,69]. Additionally, MS cellulose has emerged as a promising carrier for probiotics. Its high mechanical strength, chemical stability, and excellent biocompatibility make it an ideal protective medium for probiotics [5]. During food processing, transport, and storage, MS cellulose can effectively shield probiotics from environmental stressors such as temperature and humidity fluctuations, while gradually releasing active bacterial strains within the gastrointestinal tract [5]. This sustained release enhances probiotic colonization efficiency and biological activity, further augmenting the potential of MS and its by-products in the functional food industry.

### 6.5. Functional Lipids

MS seed oil is predominantly composed of unsaturated fatty acids (70.82%), which significantly surpasses the levels of saturated fatty acids (29.27%), highlighting its considerable potential as a functional lipid [1]. Notably, the oil’s high content of linoleic acid and alpha-linolenic acid—both well-established anti-inflammatory agents—offers distinct advantages in modulating inflammatory pathways, particularly through the inhibition of pro-inflammatory cytokines and eicosanoid synthesis. In addition, these polyunsaturated fatty acids contribute to the regulation of blood lipid profiles, exhibit antioxidant properties, and support cardiovascular health, highlighting their multifunctional bioactivity in MS-derived oil. Unsaturated fatty acids, including linoleic acid and oleic acid, are well-documented for their broad bioactive properties, such as modulating low-density lipoprotein levels and inhibiting pro-inflammatory pathways, thus conferring protective effects against metabolic disorders and cardiovascular diseases [27]. Additionally, MS seeds contain palmitic acid, a primary saturated fatty acid, which plays a role in energy metabolism and maintaining cell membrane structural integrity [1,81]. However, excessive intake of palmitic acid has been linked to an increased risk of cardiovascular diseases [82]. Therefore, the development of MS seed oil as a functional food or lipid product requires careful optimization of processing methods and fatty acid composition to achieve an optimal balance between unsaturated and saturated fatty acids, ensuring both safety and functional efficacy.

### 6.6. Potential Applications in Health Foods and Dietary Supplements

In the health food industry, the integration of MS-derived polysaccharides—some of which also contribute to dietary fiber—with other fiber components presents substantial prospects for innovation. These compounds could be developed into low-calorie sweeteners or dietary fiber supplements to address the specific dietary needs of individuals with obesity or diabetes [5,14]. Given its natural origin and notable physiological benefits, MS represents an ideal choice aligned with contemporary health-conscious dietary trends. In the realm of dietary supplements, MS extracts, owing to their broad-spectrum bioactivities, have increasingly been incorporated into complex nutritional formulations aimed at supporting overall health. MS extracts provide scientific support for enhancing bodily functions through antioxidant, anti-inflammatory, and immune-modulatory effects [51,58,68]. Noteworthy is the synergistic potential of MS when combined with other plant extracts, which enhances its comprehensive health benefits. Combining MS with other botanicals known for anti-aging or gut health-promoting properties not only broadens its application scope but also opens new avenues for the personalized development of dietary supplements [5]. This synergistic approach offers innovative solutions to a wide range of health concerns.

### 6.7. Potential Applications in Skincare and Cosmetics

MS and its by-products, enriched with a variety of bioactive compounds, exhibit remarkable antioxidant, anti-inflammatory, and moisturizing properties, making them highly promising for skincare and cosmetic formulations. These active ingredients help to delay the skin aging process, restore skin barrier function, and alleviate inflammatory responses. For instance, MS flavonoids effectively scavenge free radicals and mitigate oxidative stress, thus slowing down the skin aging process and protecting skin cells [4,6]. MS polysaccharides, with their excellent hydration capacity, are particularly suitable for developing moisturizing products such as creams and serums, significantly improving skin dryness while enhancing softness and elasticity [53].

Despite these promising properties, several challenges hinder the large-scale commercialization of MS-derived ingredients in cosmetics. One major obstacle is the variability in the concentration and composition of active compounds, which can fluctuate due to differences in geographic origin, seasonal changes, and cultivation practices. Such inconsistency may affect product efficacy and complicate quality control. Furthermore, the extraction and purification of high-value compounds like flavonoids and polysaccharides from MS often involve complex and costly processes, limiting scalability and economic feasibility. Regulatory constraints also present hurdles, as safety assessments and approval pathways for novel botanical ingredients differ across regions and can delay market entry. Addressing these challenges through standardized cultivation protocols, cost-effective extraction technologies, and harmonized regulatory frameworks will be critical for unlocking the full potential of MS in the cosmetics industry.

### 6.8. MS in Agricultural and Ecological Applications

#### 6.8.1. Fungicides and Insecticides

MS extracts, rich in secondary metabolites, exhibit notable fungicidal and insecticidal activities, positioning them as viable candidates for the development of natural plant protectants [1]. Studies have demonstrated that MS flavonoids and phenolic acids possess broad-spectrum antibacterial properties, effectively inhibiting the growth of a variety of pathogenic microorganisms (Figure 4C). For example, they significantly suppress the proliferation of plant pathogens such as *Xanthomonas campestris* and *Pseudomonas syringae*, as well as human pathogens like *Staphylococcus aureus* and *Escherichia coli* [1,83]. Additionally, MS polysaccharides and saponins exhibit inhibitory effects against fungal growth, including *Fusarium oxysporum* and *Aspergillus niger*, providing a solid scientific foundation for developing natural, safe, and environmentally friendly plant fungicides [83]. Specifically, the saponins in MS significantly inhibit insect larvae growth and reduce adult insect survival rates [84]. Compared to traditional chemical insecticides, MS extracts offer environmentally friendly advantages, with no residual risks, making them a sustainable alternative for pest management in agriculture.

#### 6.8.2. Feed Additives

MS extracts, with their diverse bioactive properties, offer broad applications in the field of animal feed additives (Figure 4D). By improving gut health, modulating immune responses, and enhancing antioxidant capacity, MS extracts significantly promote animal growth and overall health. For instance, studies have shown that MS extracts stimulate the proliferation of beneficial gut microbiota in carp, optimizing intestinal microecological balance, and improving feed conversion efficiency and weight gain [2]. Additionally, antioxidant compounds such as flavonoids and polyphenols effectively neutralize free radicals, mitigating oxidative stress-induced tissue damage and enhancing disease resistance and survival rates in animals [4]. Importantly, the saponins and other secondary metabolites in MS exhibit strong anti-inflammatory and antibacterial activities, providing a natural, environmentally sustainable alternative to antibiotics, thereby fostering the development of sustainable aquaculture practices [2,4]. Furthermore, MS extracts have been shown to regulate metabolic enzyme activity and optimize blood physiological parameters, reducing unnecessary fat deposition and energy waste in feed [2]. This feature not only improves the quality of animal meat, increasing the lean meat ratio but also enhances the overall economic efficiency of animal husbandry.

### 6.9. MS in Environmental Protection Applications

MS champ cellulose, with its renewability, cost-effectiveness, and environmental benefits, holds significant promise for environmental protection applications. As an efficient adsorbent material, MS cellulose can be employed to remove heavy metal ions, organic pollutants, and dyes from water. For instance, chemically modified or nanosized MS cellulose demonstrates increased surface area and adsorption capacity, enabling it to effectively remove contaminants such as heavy metals (e.g., lead and cadmium) and methylene blue dyes from water, providing a viable solution for water pollution control [85]. Moreover, MS cellulose holds substantial promise in the development of biodegradable materials. Biodegradable films and packaging materials derived from MS cellulose offer an alternative to conventional plastic products, reducing the accumulation of non-degradable plastics and helping alleviate plastic pollution [85,86]. This eco-friendly, sustainable approach aligns with the growing demand for green and circular economic solutions, further highlighting the potential of MS cellulose in environmental protection.

## 7. Conclusions and Future Perspectives

This review has summarized the research progress on the nutritional composition, functional components, biological activities, and safety evaluation of *Millettia speciosa* (MS) and its by-products, with a detailed discussion of their practical applications in the fields of food, medicine, and agriculture. Despite the extensive body of research, several key issues remain to be addressed to ensure the sustainable development of the MS industry. First, the bioactive components present in various parts of MS (such as leaves, flowers, seeds, and Champ) have not been fully identified. Further studies are needed to explore the interactions among these components and elucidate their mechanisms of action, enabling a comprehensive assessment of their pharmacological effects and health benefits. Second, to ensure the stability and safety of MS by-products in practical applications, there is an urgent need to strengthen standardized production processes and quality control systems. Additionally, given the variability in MS’s performance under different growing conditions, future research should focus on the impact of cultivation practices on the quality and functional components of MS. Finally, due to the wide range of applications of MS across multiple sectors, fostering interdisciplinary collaboration will be essential for advancing its translational applications in food, medicine, and agriculture. Future research in these areas will be crucial for promoting the healthy and sustainable development of the MS industry.

## Figures and Tables

**Table 3:** Potential biological activities of MS and its by-products.

Part	Assays	Biological Activity	Experimental Model	Main Findings	References
Rhizomes and roots	In vitro	Antioxidant effects	In vitro antioxidant assays	The extracts of the rhizomes and roots of MS exhibited significant antioxidant activities in vitro, including DPPH radical scavenging, ABTS radical scavenging, and ferric reducing antioxidant power (FRAP) assays. The antioxidant activity of the rhizome extracts was significantly higher than that of the root extracts	[52]
Champ	In vitro	Antioxidant effects	In vitro antioxidant assays	The modified polysaccharide from MS Champ exhibits significant antioxidant activity. The modified polysaccharide shows strong free radical scavenging ability in both DPPH and ABTS free radical scavenging assays.	[53]
Champ	In vitro	Antioxidant effects	In vitro antioxidant assays	Flavonoid extracts obtained using different extraction methods (ultrasound, microwave, and Soxhlet extraction) all exhibited significant antioxidant activity. In the DPPH radical scavenging assay, the microwave extract showed the highest scavenging capacity (IC50 = 0.34 mg/mL), followed by the ultrasound extract and Soxhlet extract. Additionally, in the ABTS radical scavenging assay and the total antioxidant capacity (FRAP) test, the microwave extract also demonstrated the strongest activity.	[54]
Leaves	In vitro	Antioxidant effects	In vitro antioxidant assays	The leaf extract of MS exhibits significant antioxidant activity. The DPPH radical scavenging assay results show that the extract has good scavenging ability. The ABTS radical scavenging assay and reducing power test (FRAP) also demonstrate that the extract shows strong antioxidant effects. Additionally, the study found that the contents of polysaccharides, flavonoids, and saponins in the extract are positively correlated with its antioxidant activity.	[55]
Champ	In vivo	Regulation of lipid metabolism	C57BL/6 Mice	The flavonoid extract MS Champ combats obesity by promoting thermogenesis in brown adipose tissue, increasing fatty acid oxidation, inhibiting fat accumulation, and regulating the expression of genes related to lipid metabolism. This results in a reduction in body weight and body fat content in mice, as well as an improvement in their lipid metabolic indicators.	[8]
Champ	In vivo	Regulation of lipid metabolism	Mice	The fermented beverage of MS Champ directly activates PKA/PKG-mediated kinase-sensitive hormone-sensitive lipase (p-HSL) through the cAMP signaling pathway, promoting fat breakdown and thus combating obesity.	[56]
Seed	In vitro	Anti-bacterial effect	In vitro Anti-bacterial assays	The MS seed extract can significantly inhibit the growth of five Gram-negative strains (*Ralstonia solanacearum*, *Agrobacterium tumefaciens*, *Xanthomonas vesicatoria*, *Pseudomonas lachrymans*, and *Escherichia coli*) and two Gram-positive strains (*Staphylococcus haemolyticus* and *Bacillus subtilis*).	[1]
Leaves	In vitro	Anti-bacterial effect	In vitro Anti-bacterial assays	The MS Leaves extract can significantly inhibit the growth of five Gram-negative strains (*Ralstonia solanacearum*, *Agrobacterium tumefaciens*, *Xanthomonas vesicatoria*, *Pseudomonas lachrymans*, and *Escherichia coli*) and two Gram-positive strains (*Staphylococcus haemolyticus* and *Bacillus subtilis*).	[1]
Flower	In vitro	Anti-bacterial effect	In vitro Anti-bacterial assays	The MS Flower extract can significantly inhibit the growth of five Gram-negative strains (*Ralstonia solanacearum*, *Agrobacterium tumefaciens*, *Xanthomonas vesicatoria*, *Pseudomonas lachrymans*, and *Escherichia coli*) and two Gram-positive strains (*Staphylococcus haemolyticus* and *Bacillus subtilis*).	[1]
Champ	In vivo	Anti-bacterial effect	Mice	The extract of MS regulates glycolipid metabolism, lowers blood glucose levels, improves insulin resistance, and alleviates the metabolic disorders induced by diabetes through the modulation of related metabolic pathways.	[10]
Champ	In vivo	Anti-inflammatory activity	Mice	The polysaccharides from MS Champ can alleviate the symptoms of ulcerative colitis induced by dextran sulfate sodium (DSS), significantly reduce the level of intestinal inflammation, decrease pathological damage to the colon tissue, and inhibit the expression of inflammation-related factors.	[57]
Champ	In vivo	Anti-inflammatory activity	Mice	MS champ polysaccharides improve gut microbiota and inhibit inflammatory responses.	[58]
Champ	In vivo	Anti-inflammatory activity	Mice	MS champ polysaccharides inhibit the expression of pyroptosis-related factors by suppressing the caspase-1/gasdermin D/interleukin-1β signaling pathway, reducing the inflammatory response, and further alleviating tissue damage caused by inflammation.	[59]
Champ	In vivo	Antifatigue	Mice	MS extract improves endurance, extends swimming time, and alleviates fatigue symptoms in mice by reducing blood lactate levels and increasing liver glycogen reserves.	[2]
Champ	In vivo	Antifatigue	Mice	The polysaccharides from MS champ can enhance the exercise endurance of mice, prolong swimming time, and significantly reduce blood lactate levels.	[60]
Champ	In vivo	Antifatigue	Mice	*MS* champ polysaccharides effectively alleviated fatigue symptoms caused by excessive exercise by enhancing the endurance of mice, prolonging swimming time, reducing blood lactate levels, and increasing liver glycogen reserves.	[61]
Champ	In vitro	Regulation of immune function	Cyprinus carpio	The extract of MS champ regulates the expression of immune-related factors, improves the immune response in cyprinus carpio, and enhances resistance to pathogenic microorganisms.	[2]
Champ	In vitro	Regulation of immune function	RAW.264 cells	The polysaccharide can enhance the phagocytic function of macrophages, promote the proliferation of leukocytes, regulate the secretion of cytokines by immune cells, and improve the overall function of the immune system.	[17]
Champ	In vivo	Improves of intestinal homeostasis	Mice	The polysaccharides from MS can enhance intestinal barrier function, promote the growth of beneficial microbiota, and inhibit the proliferation of harmful bacteria, thereby improving gut health.	[18]
Champ	In vitro	Regulation of immune function	RAW.264 cells	MS polysaccharides can significantly enhance the phagocytic ability of macrophages, increase the proliferation rate of leukocytes, and promote immune system activity by regulating the secretion of cytokines.	[53]
Champ	In vivo	Regulation of immune function	Mice	MS champ polysaccharides can alleviate cyclophosphamide-induced immunosuppression by improving gut microbiota balance and enhancing immune system function, demonstrating significant immunoregulatory effects.	[60]
Champ	In vivo	Improves of intestinal homeostasis	Mice	The polysaccharides from MS champ alleviate intestinal inflammation induced by dextran sulfate sodium (DSS), significantly reduce the level of inflammation in the gut, decrease pathological damage in colon tissue, and inhibit the expression of inflammatory factors, thereby providing protective effects on intestinal health.	[57]
Champ	In vivo	Anti-depression	Mice	The extract of MS champ significantly improved the metabolic levels in depressed rats, reducing the changes in metabolites associated with depression, possibly by regulating the synthesis of neurotransmitters and energy metabolism.	[61]

**Table 4:** In vivo safety evaluation and toxicological description of MS and its byproducts.

Research Type	Research Object	Visual Object	Intervention Time	Dose	Safety Evaluation Description	References
Cytotoxic effect	L929 cells	CCK-8	24 h	50 μg/mL 10 μg/mL 50 μg/mL 200 μg/mL	The extract of MS seeds exhibits good selective cytotoxicity within a certain dosage range and demonstrates high safety for normal cell	[1]
Cytotoxic effect	L929 cells	CCK-8	24 h	50 μg/mL 10 μg/mL 50 μg/mL 200 μg/mL	The extract of MS Leaves exhibits good selective cytotoxicity within a certain dosage range and demonstrates high safety for normal cell	[1]
Cytotoxic effect	L929 cells	CCK-8	24 h	50 μg/mL 10 μg/mL 50 μg/mL 200 μg/mL	The extract of MS flower exhibits good selective cytotoxicity within a certain dosage range and demonstrates high safety for normal cell	[1]
Animal study	Mice	Weight changes, Clinical symptoms	-	-	The given dose of MS Radix extract, no significant acute toxic reactions were observed in the clinical symptoms of mice.	[4]
Animal study	Mice	Blood biochemical indicators Histopathology	14 days	2 g/(kg·bw) 5 g/(kg·bw)	After a single high-dose administration of MS champ extract, no animal deaths were observed, and the LD50 (median lethal dose) was found to be greater than 5 g/kg, indicating low acute toxicity.	[75]
Animal study	Mice	Blood biochemical indicators, Histopathology	90 days	0.5 g/(kg·bw) 1 g/(kg·bw) 2 g/(kg·bw)	MS champ extract did not cause significant effects on body weight gain, hematological indicators, blood biochemical markers, or the histopathology of major organs.	[75]
Animal study	Mice	Body weight, Hematological parameters, Biochemical indicators, Histopathological changes of major organs	90 days	0.86 g/(kg·bw) 2.58 g/(kg·bw) 7.73 g/(kg·bw)	After administration of MS extract, no obvious toxic reactions were observed in the mice’s body weight, hematological parameters, and biochemical indicators, and no significant abnormalities were found in any of the indicators.	[76]
Animal study	Mice	Body weight, Hematological parameters, Biochemical indicators, Histopathological changes of major organs	-	-	No significant acute toxic reactions were observed with the given dose of MS leaf extract.	[77]

## Data Availability

No new data were created or analyzed in this study. Data sharing is not applicable to this article.
